# Efficacy and toxicity of vemurafenib and cobimetinib in relation to plasma concentrations, after administration via feeding tube in patients with *BRAF*-mutated thyroid cancer: a case series and review of literature

**DOI:** 10.1007/s00280-022-04437-z

**Published:** 2022-05-22

**Authors:** J. M. van Berge Henegouwen, H. van der Wijngaart, L. J. Zeverijn, L. R. Hoes, M. Meertens, A. D. R. Huitema, L. A. Devriese, M. Labots, H. M. W. Verheul, E. E. Voest, H. Gelderblom

**Affiliations:** 1 Department of Medical Oncology, Leiden University Medical Center, Leiden, The Netherlands; 2 Department of Medical Oncology, Amsterdam University Medical Center, Vrije Universiteit Amsterdam, Cancer Center Amsterdam, Amsterdam, The Netherlands; 3 Department of Molecular Oncology and Immunology, Netherlands Cancer Institute, Amsterdam, The Netherlands; 4 Department of Pharmacy and Pharmacology, Netherlands Cancer Institute, Amsterdam, The Netherlands; 5 Department of Pharmacology, Princess Maxima Center for Pediatric Oncology, Utrecht, The Netherlands; 6 Department of Clinical Pharmacy, University Medical Center Utrecht, Utrecht, The Netherlands; 7 Department of Medical Oncology, University Medical Center Utrecht, Utrecht, The Netherlands; 8 Department of Medical Oncology, Radboud University Medical Center, Nijmegen, The Netherlands

**Keywords:** Vemurafenib, Cobimetinib, Thyroid carcinoma, Feeding tube, Plasma concentrations, Case series

## Abstract

**Introduction:**

The combination of vemurafenib, a proto-oncogene B-Raf inhibitor (BRAFi) and cobimetinib, an inhibitor of mitogen-activated protein kinase kinase (MEKi) has shown to improve survival in patients with *BRAF* V600-mutated melanoma. *BRAF* mutations are also frequently detected driver mutations in other tumor types, including thyroid carcinoma. Since thyroid carcinoma is not a labeled indication for BRAF/MEKi, a cohort for patients with *BRAF* V600-mutated thyroid carcinoma was opened within the Drug Rediscovery Protocol (DRUP), a national ongoing pan-cancer multi-drug trial, in which patients receive off-label treatment with approved drugs based on their molecular tumor profile.

**Results:**

Here, we present two patients with *BRAF*-mutated thyroid carcinoma, who were successfully treated with vemurafenib/cobimetinib administered via a feeding tube. Plasma concentrations of vemurafenib and cobimetinib were determined. A partial response was observed in both patients, but they experienced significant toxicity.

**Conclusion:**

Our cases show that vemurafenib/cobimetinib treatment is effective in *BRAF* V600-mutated thyroid carcinoma, also when administered via a feeding tube. Although serious side effects occurred in both patients, we hypothesize that this was not attributable to the administration route. Therefore, administration of vemurafenib/cobimetinib by feeding tube is feasible and effective.

**Trial registration:**

Clinical trial identification: NCT02925234.

## Introduction

The development of a large number of targeted- and immunotherapies, targeting specific molecular alterations and aberrant signaling pathways in tumor cells, has dramatically changed the treatment paradigm in oncology in the past decade [1]. Coming from a histology-centered approach in systemic treatment of patients with cancer, focus has now shifted to a patient-centered biomarker-driven approach [1]. Many targeted- and immunotherapies have already received FDA/EMA approval and are available for patients with multiple well defined tumor types, harboring a specific molecular feature that predicts drug sensitivity [2–6].

However, due to histology-specific registrations of these drugs, a significant number of patients with other tumor types harboring similar qualifying genomic aberrations do not have access to these potentially active therapies. In the Drug Rediscovery Protocol (DRUP) [7], a national ongoing pan-cancer multi-drug basket/umbrella trial, patients are treated off-label with registered drugs based on their tumor molecular profile. The innovative design allows for an infinite number of cohorts, testing multiple hypotheses in parallel. The DRUP facilitates access to potentially effective drugs for patients with a tumor with a specific molecular profile, while systematically collecting clinical data on efficacy and safety of these drugs when used off-label. As part of the trial, whole-genome sequencing is performed on fresh tumor biopsies at baseline for biomarker analysis [7].

The combination of vemurafenib, an inhibitor of proto-oncogene B-Raf (BRAF), and cobimetinib, an inhibitor of mitogen-activated protein kinase kinase (MEK) is one of the available treatment options in DRUP. BRAF and MEK inhibitor combinations (BRAF/MEKi) have impressively improved the survival of patients with stage IV melanoma harboring a *BRAF* V600 mutation [6]. *BRAF* mutations are also frequent driver mutations in other tumor types, such as colorectal cancer (13.1%) and non-small cell lung cancer (5.6%) [8]. Moreover, *BRAF* mutations are the most common genetic alteration in thyroid cancer, occurring in 60% of patients with papillary thyroid cancer and in 29% of patients with anaplastic thyroid cancer (ATC) [9–13]. Since thyroid cancer is not a labeled indication for BRAF/MEKi, a cohort for patients with *BRAF* V600-mutated thyroid cancer was opened in DRUP.

Two patients in this cohort had difficulties swallowing the tablets due to the localization of the tumor and prior local treatment. These patients were, by exception to the protocol, allowed to take their medication by feeding tube. Remarkably, both patients experienced toxicity upon treatment, for which a relationship with the administration route could not fully be ruled out. Here, we present these two cases, including plasma concentrations of both drugs, as a learning opportunity for other physicians and pharmacists involved in the individualized treatment of patients with targeted therapies.

## Case presentation

### Patient 1

A 71-year-old female patient presented with difficulties swallowing, stridor, and progressive shortness of breath. A thyroid tumor, compressing the trachea, was detected. Tumor histopathology revealed the presence of a double tumor; a *BRAF* V600-mutated T4aN1bM0 papillary thyroid carcinoma and a squamous cell carcinoma of yet unknown primary origin, both located in the thyroid gland. Because of its locally advanced nature with the airway, (emergency) tracheostomy was performed, followed by radiotherapy of the neck area and I-131 treatment. Due to dysphagia, as a consequence of the extensive surgery and radiation, a percutaneous endoscopic gastrostomy (PEG) tube was inserted for intake. After completion of standard I-131 treatment for the papillary thyroid carcinoma, follow-up PET-CT scan was performed, which revealed multiple FDG positive lung metastases. Molecular evaluation of the lung metastases showed the presence of the same *BRAF* V600 mutation, indicating clonal relationship with the thyroid tumor. Therefore, the diagnosis was pulmonary metastases of a primary *BRAF* V600-mutated squamous cell thyroid carcinoma.

The patient was thereafter included in the DRUP trial and received vemurafenib/cobimetinib combination treatment, targeting the *BRAF* mutation. Because of the dysphagia, medication had to be administered via the feeding tube. After consultation with the trial pharmacist and with agreement of the study team, vemurafenib and cobimetinib tablets were disintegrated in 30 mL water (~ 35 °C) in a 50 mL syringe, and administered via the feeding tube, which was flushed with 20 mL of water before and after administration. Furthermore, trough concentrations (*C*_min_) were monitored to make dose adjustments if necessary. Although the patient benefited from omeprazole for pyrosis, it was advised to discontinue proton-pump inhibition.

**Fig. 1:**
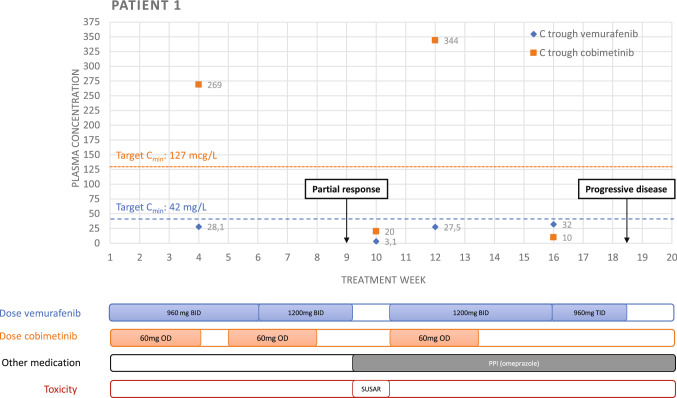
Trough plasma concentrations of vemurafenib and cobimetinib for patient 1 during the course of the treatment. Dosage and treatment interruptions, co-medication and adverse events are presented below the chart. *C*_min_ trough concentration, *OD* once daily, *BID* twice daily, *TID* thrice daily, *PPI* proton-pump inhibitor, *SUSAR* Suspected Unexpected Serious Adverse Reaction

On 8th of March 2019, the patient started with twice-daily administration of vemurafenib 960 mg and once-daily 60 mg cobimetinib via the feeding tube. After three weeks *C*_min_ was measured, revealing concentrations of 269 mcg/L for cobimetinib (target *C*_min_ 127 mcg/L) [14] and 28.1 mg/L for vemurafenib (target *C*_min_ 42 mg/L) [15] (Fig. 1). Therefore, dosage of vemurafenib was increased to 1200 mg twice daily, whereas the dosage of cobimetinib remained unchanged. After two treatment cycles the first response evaluation, using Response Evaluation Criteria in Solid Tumors (RECIST v1.1), showed a partial response with a decrease of 45% in sum of target lesions.

After two months, the patient was admitted to the hospital with a gastric perforation, localized near to the tip of the feeding tube. She was treated with intravenous pantoprazole and both vemurafenib and cobimetinib were interrupted for 2 weeks. The perforation was thought to be possibly related to study treatment. However, a mechanical component of the feeding tube had also been thought to play a role in the pathogenesis. A stool antigen test for Helicobacter pylori infection was negative. *C*_min_ measurements were performed 10 days after admission, revealing concentrations below the threshold for both treatments (vemurafenib 3.1 mg/L and cobimetinib 20 mcg/L). Since the patient clearly benefited from treatment, it was decided to restart the combination treatment without dose reduction after the patient fully recovered (on the 15th of May), together with continuation of a proton-pump inhibitor (esomeprazole granules 20 mg twice daily) and monitoring of the *C*_min_.

Two weeks after restart of the treatment, cobimetinib concentrations were far above the threshold, while vemurafenib concentrations were still below the threshold (344 mcg/L and 27.5 mg/L, respectively). Due to multiple grade 1/2 adverse events related to MEKi (fatigue, arthralgia, rash, and edema), cobimetinib was discontinued 14 weeks after treatment initiation. Despite increasing the dosage of vemurafenib to 960 mg 3 times a day, *C*_min_ remained below the target *C*_min_ in the 16th week of treatment.

The second response evaluation after four cycles showed progressive disease with an increase of 68% in sum of target lesions, following definitive discontinuation of the treatment. The patient died 6 months after discontinuation due to progressive disease. The progression-free survival was 18 weeks.

### Patient 2

A 64-year-old male, with a history of hypertension, smoking and chronic obstructive pulmonary disease, presented in May 2019 with a lump in the neck and complaints of coughing, hoarseness, and a sore throat. The patient was diagnosed with a pT4bN1bM0 ATC in the left thyroid lobe. Hemi-thyroidectomy with radical resection of the tumor, including a selective cervical lymph node dissection was performed. Pathological examination of the resected tissue revealed a 95 mm large ATC, with vaso-invasive expansion as well as expansion in the surrounding soft tissue. Five out of 18 resected lymph nodes showed intracapsular metastases.

The patient was treated with adjuvant chemoradiotherapy. As intake difficulties due to mucositis were anticipated, a prophylactic PEG-tube was placed for nutrition. Within one month after completing chemoradiotherapy, the patient was diagnosed with retrosternal, cutaneous, and pulmonary metastases. Panel-based next generation sequencing of a cutaneous lesion revealed a *BRAF* V600E mutation. Since no effective standard treatment options are available for metastatic ATC, the patient was referred for treatment within the DRUP study, and was allocated treatment with vemurafenib and cobimetinib, targeting *BRAF* V600E mutation.

**Fig. 2:**
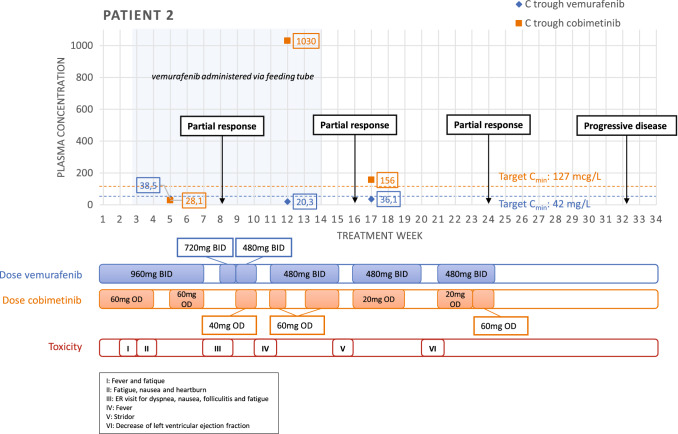
Trough plasma concentrations of vemurafenib and cobimetinib for patient 2 during the course of the treatment. Dosage and treatment interruptions and adverse events are presented below the chart. *C*_min_ trough concentration, *OD* once daily, *BID* twice daily

The patient started study treatment with vemurafenib 960 mg twice daily and cobimetinib 60 mg once daily in October 2019. After one week of treatment, the patient presented with fever, upon which the study treatment was interrupted for one day. Due to swallowing difficulties of the vemurafenib tablets, the study team allowed to disintegrate the vemurafenib tablets in 30 mL water (~ 35 °C) in a 50 mL syringe, and to administer this solution through the feeding tube. Before and after administration, the feeding tube was flushed with 20 mL of water. Furthermore, the study team advised to monitor plasma drug concentrations (Fig. 2). On day 15, he experienced grade 1 fatigue, nausea, and dyspepsia, considered to be probably related to study medication. For these adverse events he received treatment with a proton-pump inhibitor and lorazepam. *C*_min_ for both vemurafenib and cobimetinib were below the target *C*_min_; 38,5 mg/L and 28,1 mcg/L, respectively. However, given the adverse events, it was decided not to increase the doses. At the end of cycle 2, at 7 weeks, he presented with dyspnea and grade 1 nausea, vomiting, folliculitis, and grade 2 fatigue. The study treatment was interrupted for five days. Two days after re-introduction of the study treatment, he presented again with grade 2 fatigue, nausea, and dyspnea, probably related to the study medication, for which the doses of both drugs were reduced to 720 mg twice daily for vemurafenib and 40 mg once daily for cobimetinib, with limited effect (Fig. 2).

The first tumor evaluation after eight weeks showed a partial response, with 50% decrease of volume of target lesions. At one week after the start of cycle 3, he experienced a fever, for which the study treatment was interrupted during five days. After re-introduction of the treatment (vemurafenib 480 mg BID, cobimetinib 60 mg OD), tolerance improved. During cycle 4, the treatment was interrupted for five days because of an inspiratory stridor, which was treated with dexamethasone and antibiotics. After 14 weeks of treatment, the esophageal pain decreased and was the patient able to take all study medication orally again. In response to the extremely high plasma trough level of cobimetinib (1030 mcg/L, versus population mean of 127 mcg/L), the dose of cobimetinib was reduced to 20 mg daily.

A routine check of cardiac function during cycle 5 showed a significant decrease of left ventricular ejection fraction to 39%, compared to 62% at baseline, considered to be probably related to cobimetinib. The study medication was interrupted for a week. At the end of cycle 6, the patient presented with a rash, possibly related to the study medication. Because of multiple recurring toxicities, it was decided to discontinue the treatment. Within 3 months left ventricular ejection fraction recovered and the patient experienced a good quality of life then. Two months after terminating the study therapy, multiple new metastatic lesions were discovered showing progressive disease. The progression free survival was 32 weeks.

## Discussion

Our cases demonstrate that a combination of the BRAFi vemurafenib and the MEKi cobimetinib is feasible when administered via feeding tube. For both patients included in the current paper a significant decrease in tumor volume was reached. However, both patients experienced significant toxicity upon treatment.

Our efficacy findings are in line with previous reports on oral treatment with BRAF inhibitors in *BRAF* V600-mutated anaplastic and papillary thyroid cancer, in which efficacy of treatment was reported. For instance, Brose et al. observed a partial response in 16 of 48 patients (33%) with metastatic radioactive iodine refractory *BRAF* V600 papillary thyroid cancer treated with vemurafenib monotherapy [17]. In an open-label phase II trial, a response rate of 69% was achieved in *BRAF* V600-mutated ATC patients treated with dabrafenib (BRAFi) and trametinib (MEKi) [18].

In other types of cancer, several reports have been published on the efficacy of different oral targeted anti-cancer agents, including tyrosine kinase inhibitors (TKIs), when administered via a feeding tube. However, the majority of the available data on alternative administration routes comprises single case reports [19–22]. Moreover, only a few prospective studies focused on the bioavailability of these compounds when administered via a nasogastric or PEG tube. Using this administration route, Cantarini et al. described the pharmacokinetic patterns of the EGFR TKI gefitinib in healthy volunteers, and Chiu et al. studied the pharmacokinetics of dacomitinib (pan-HER TKI) in patients with locally advanced head and neck squamous cell carcinoma [23, 24]. Although Cantarini et al. observed no differences in systemic exposure between oral administration or nasogastric feeding tube administration in healthy participants, Ciu et al. observed a 34% reduction in *C*_max_ in patients who were treated via the feeding tube, which could imply that only a subset of developed agents could be administered in an efficient way using a nasogastric- or PEG tube.

Over the past decade, only a few reports of alternative administration of vemurafenib were published. Khimani et al. were one of the first who described successful vemurafenib treatment via feeding tube in a patient with *BRAF* V600 -mutated melanoma [25]. Interestingly, this is the first case description of alternative administration of cobimetinib. Moreover, this report is the first report collecting therapeutic drug monitoring data during alternative administration of both drugs. In our two cases, trough plasma concentrations of vemurafenib either did not reach or hardly reached the target *C*_min_, while cobimetinib trough concentrations were relatively high. For patient 2, we have observed a high variability of cobimetinib plasma concentrations. The first measurement was performed during the prescribed cobimetinib treatment break of 7 days, explaining the concentration below the target *C*_min_. However, the extreme concentration of 1030 mcg/L of the second measurement remained unexplained. Therefore, interpatient variability of drug concentration and sensitivity should also be taken into account when interpreting *C*_min_.

Cobimetinib is a Biopharmaceutical Classification System (BCS) class I compound indicating both good water solubility and permeability. Therefore, alternative methods of administration, e.g., using feeding tubes, are not expected to highly influence absorption, which is in accordance with our observations in these two patients. Vemurafenib is a BCS class IV compound characterized by both low solubility and low permeability. To increase bioavailability, vemurafenib is formulated as a solid dispersion tablet with favorable dissolution characteristics [26]. By crushing the tablets (disintegration) prior to administration, the solid dispersion is damaged which may have major impact on the solubility at the site of absorption and consequently on bioavailability. Here, we show that despite these unfavorable characteristics, it is still possible to achieve relevant systemic exposure in patients. Although target *C*_min_ for vemurafenib and cobimetinib in thyroid cancer remains unknown, it is expected that these targets are within the same range as for melanoma [16]. Notably, vemurafenib exposure of case 2 seemed to be comparable during administration via feeding tube and oral administration (38.5 and 20.3 vs. 36.1 mg/L), suggesting only marginal effect of the administration route on plasma drug concentrations. Still, therapeutic drug monitoring to manage administration of vemurafenib via feeding tube could be useful to optimize treatment.

Of importance, both patients experienced serious side effects during treatment. Patient 1 was admitted to the hospital because of a gastric perforation, occurring after 2 months of treatment. The administration route of medication might have contributed to this event. Additionally, the low solubility of vemurafenib may have played a role in either developing or worsening gastrointestinal toxicity, as undissolved particles could deposit on the gastrointestinal mucosa. On the other hand, gastrointestinal perforations have been described during MEKi therapy [27]. In case 2, a considerable decrease in left ventricular function was observed after 4 months of treatment, which is a known adverse event from BRAF/MEKi treatment (Risk Ratio: 2.79; 95% CI 1.36–5.73) [28]. Interestingly, this patient only received vemurafenib via the feeding tube and the administration route could thus not have contributed to the degree of toxicity. Nevertheless, the extremely high concentration of cobimetinib might have worsened the extent of the toxicity (Fig. 2).

Taken together, this case series demonstrates the challenges that patients and practitioners face when using alternative administration routes of novel anti-cancer drugs. Up to now, only a few systematic prospective studies have been carried out studying the bioavailability of new anti-cancer drugs when administered via feeding tube. However, in a significant subset of cancer patients, especially patients with cancer in the head and neck region, a nasogastric or PEG-tube is essential for sufficient enteral nutrition. Both of our patients showed clinical benefit with a partial response, but experienced clinically significant toxicity. Since similar side effects have been observed in patients who received the included treatments orally, it is not likely that the observed toxicity was completely attributable to the administration route. However, a causal relationship cannot be ruled out. Nonetheless, we do believe that vemurafenib/cobimetinib administration via feeding tube can successfully be performed, if no other alternatives are available. Our findings indicate that therapeutic drug monitoring should be part of patient management when these drugs are administered via feeding tubes, given the intra-individual variability in drug exposure and clinical impact.
